# Circadian Fatigue or Unrecognized Restless Legs Syndrome? The Post-Polio Syndrome Model

**DOI:** 10.3389/fneur.2014.00115

**Published:** 2014-07-07

**Authors:** Andrea Romigi, Michelangelo Maestri

**Affiliations:** ^1^Neurophysiopathology Unit, Department of Systems Medicine, Sleep Medicine Centre, Tor Vergata University and Hospital, Rome, Italy; ^2^IRCCS Neuromed, Pozzilli, Italy; ^3^Neurology Unit, Department of Clinical and Experimental Medicine, University of Pisa, Pisa, Italy

**Keywords:** restless legs syndrome, post-polio syndrome, fatigue, sleep, PLMS

“It ought to be generally known that the source of our pleasure, merriment, laughter, amusement, as of our grief, pain, anxiety, and tears, is none other than the brain”

–Hippocrates

The discussion of this research paper may represent the starting point of the debate about the influence of sleep disorders on fatigue perception. These authors observed in a small cohort of patients with paralytic poliomyelitis (PP) and post-polio syndrome (PPS) a significant circadian variation of subjective fatigue evaluated by means of a validated scale. These authors showed a progressive worsening of fatigue during afternoon in both PP and PPS (1). Polysomnographic variables did not correlate with fatigue, albeit restless legs syndrome (RLS) symptoms were not evaluated. PP is an acute poliovirus infection resulting in flaccid paralysis, due to poliovirus-induced apoptosis, and consequent central nervous system injury, which leads to paralysis. Therefore, during its clinical course an increasing and progressive fatigue may represent an integral part of PP motor symptoms. In addition, more than 90% of patients with PP develop a delayed syndrome characterized by excessive fatigue (2). PPS circadian impairment of fatigue may be more puzzling. It may be related to the presence of sleep disorders [i.e., sleep apnea, RLS, and periodic limb movements of sleep (PLMS)] previously described in PPS (3–5).

Restless legs syndrome and other sleep disorders have been reported in small PPS uncontrolled cohorts or single case reports, and in selected samples complaining of fatigue and sleepiness. In addition, fatigue represents a cardinal symptom of PPS, marked by a progressive course, and able to affect significantly patient quality of life (6). Moreover, fatigue in PPS may be characterized by circadian changes, as recently showed by Viana and colleagues in a small cohort of PPS patients (1). Although, these authors did not explore clinical RLS in these PPS sample, it is intriguing to note that fatigue could resemble the circadian pattern of RLS symptoms. Therefore, RLS may represent an interesting model and a possible unifying hypothesis for fatigue in PPS similarly to other diseases both neurological (i.e., multiple sclerosis, myotonic dystrophies) and non-neurological (COPD, liver disorders) (2, 7–9). Prevalence of RLS in these disorders has been reported as higher than in the general population, but could even be underdiagnosed because of difficulties to distinguish from other symptoms such as pain or spasticity.

Two different pathogenic hypotheses are interesting to highlight in this context. First, more than 90% of neurological conditions associated with RLS are linked to inflammatory mechanisms that have been supposed to play a role also in primary RLS (10, 11). On the other side, there is growing evidence that the fatigue is related to the interplay between neurotransmitters and immunological imbalance and deranged hypothalamic–pituitary–adrenal axis in states of chronic stress. In addition, fatigue affects several CNS regions and pro-inflammatory cytokines (i.e., IL-1, IL-6, and TNF-alpha) that are released during systemic inflammation and are activated by reduced corticotropin releasing factor and low cortisol concentrations (2, 12). This hypothesis seems to accord with experimental data that in immune-mediated disorders, acute stress can be beneficial because rises in corticotropin releasing factor attenuate the T-helper-1 cell response. However, it is still unclear if chronic stress versus acute stress down regulates the hypothalamic–pituitary–adrenal axis, leading to persistent fatigue. This axis is underactive in patients with chronic fatigue syndrome, post-traumatic stress disorder, fibromyalgia, and also in PPS (2). In addition, it has not been clarified if the PPS denervation processes are caused by further loss of motor neurons due to normal aging or active chronic inflammatory processes as supported by the increased expression of pro-inflammatory cytokines (13). Therefore, both diseases may share a pro-inflammatory condition. On the other side, an alternative explanation for the supposed link between RLS and PPS should consider that the sequelae of polio, similarly to other spinal cord RLS-related diseases, may lead to abnormal sensorimotor integration at the spinal interneuronal level, probably mediated by dopaminergic deficit, and/or to loss of supraspinal inhibitory influences (14–16). The diencephalon–spinal dopaminergic pathway, which projects its terminal innervations in the dorsal horn at all spinal levels (16), may represent a hypothetical common anatomical substrate involving RLS and PPS.

Viana et al. found a circadian trend of fatigue in PPS, but they did not evaluate RLS symptoms or potential association of PLMS with disturbed sleep although they showed pathological PLMS in more than one third of PPS as reported elsewhere (4). Therefore, we conjecture that a circadian variation of fatigue may be related to unrecognized RLS symptoms in PPS previously reported in few PPS cases (3, 5). Although small PPS samples with RLS and/or PLMS does not allow any generalization, the common inflammatory pathways and also a natural propensity to circadian trend of fatigue in PPS, as interestingly demonstrated by Viana et al. (1), may support the hypothesis of a secondary RLS in PPS. Epidemiologic studies with controlled design are needed to explore the real magnitude of RLS in PPS besides in other neurological diseases and to evaluate possible common mechanisms involved in the development of fatigue and RLS. In a more general context, the interplay between fatigue and RLS should be further evaluated in both primary and “secondary” form, also applying more objective tools such as periodic limb movements or response to dopaminergic therapy and also considering confounding factors such as depression or sleep related breathing disorders.

## Conflict of Interest Statement

The authors declare that the research was conducted in the absence of any commercial or financial relationships that could be construed as a potential conflict of interest.
